# Evaluation of a chromogenic medium, CHROMagar StrepA, for the detection of *Streptococcus pyogenes* in throat swab samples

**DOI:** 10.1128/spectrum.00050-26

**Published:** 2026-05-15

**Authors:** Romane Ponthot, Koukab Mahrous, Alexandre Grimmelprez, Patricia Schatt

**Affiliations:** 1 Microbiology Department, Laboratoire Clinique Notre-Dame de Grâce (CNDG) 82247 , Gosselies, Belgium; Creighton University, Omaha, Nebraska, USA

**Keywords:** diagnostic microbiology, rapid detection, throat culture, chromogenic medium, CHROMagar StrepA, *Streptococcus pyogenes*

## Abstract

*Streptococcus pyogenes* is a common bacterium that causes a wide spectrum of diseases, ranging from mild pharyngitis to life-threatening invasive infections. Rapid and accurate detection is essential for optimal patient management. In this study, the performance of a chromogenic medium, CHROMagar StrepA, was evaluated for the detection of *S. pyogenes* in throat swab samples. The evaluation was conducted in three successive phases. Phase 1 involved testing using pure bacterial isolates. Phase 2 consisted of a semi-quantitative comparison with conventional culture on COL-S medium (Columbia agar supplemented with sheep blood). Phase 3 assessed diagnostic performance in routine clinical practice, in parallel with a rapid antigen detection test (OSOM Strep A Test). Diagnostic performance metrics, including sensitivity, specificity, positive predictive value, and negative predictive value, were calculated. CHROMagar StrepA demonstrated 100% specificity and sensitivity for the detection of *S. pyogenes* when compared with conventional culture. It enables reliable, visually distinctive identification of *S. pyogenes* in culture, although certain non-Group A *Streptococcus* (GAS) streptococcal strains remain challenging to detect. These results support the use of CHROMagar StrepA as a culture-based approach for detecting *S. pyogenes*, while identifying limitations in the detection of non-GAS streptococci and selectivity that may require further optimization.

**IMPORTANCE:**

Accurate identification of *Streptococcus pyogenes* is essential for the appropriate management of patients with sore throat and for preventing complications. While rapid tests provide quick results, they may miss infections when bacterial levels are low, making culture methods still necessary in many cases. This study evaluates a chromogenic culture medium designed to simplify the detection of *S. pyogenes* by producing easily recognizable colonies. By improving visual interpretation and reducing reliance on technical expertise, this approach may help clinical laboratories work more efficiently and confidently. The findings provide practical insight into the strengths and limitations of a chromogenic medium compared with conventional culture and rapid testing, supporting informed decisions about its use in routine diagnostic practice.

## INTRODUCTION

Modern medicine is continually advancing, particularly in infectious disease diagnostics, where rapid and reliable pathogen detection is critical. *Streptococcus pyogenes* (Group A Streptococcus, GAS) is a significant human pathogen and the primary bacterial cause of pharyngitis, accounting for 15%–30% of pediatric and 5%–10% of adult cases, although most cases are viral (1). Accurate identification is essential to ensure appropriate antimicrobial therapy and prevent post-infectious complications such as acute rheumatic fever and glomerulonephritis (2, 3).

Current diagnostic approaches include rapid antigen detection tests, PCR-based assays, and conventional culture on blood-based media (3, 4). While rapid tests offer short turnaround times, their sensitivity may be suboptimal, particularly in specimens with low bacterial load. Culture-based methods remain the reference standard but require additional incubation and skilled interpretation, especially when differentiating GAS from other beta-hemolytic streptococci.

To address these limitations, CHROMagar StrepA (CHROMagar, Kanto Chemical Group, Paris, France) was developed as a chromogenic medium that allows rapid visual identification of GAS through distinctive colony coloration.

The objective of this study was to evaluate the diagnostic performance of CHROMagar StrepA compared with conventional culture methods and rapid antigen testing, focusing on sensitivity, specificity, and practical utility, as well as its potential integration into routine clinical microbiology workflows.

## MATERIALS AND METHODS

The exact formulation of CHROMagar StrepA is proprietary. However, it includes a powder base (54.3 g/L), one liquid supplement (S1: 2 mL/L), and one powder supplement (S2: 0.1 g/L). The base medium contains agar, peptones, yeast extracts, salts, growth factors, and chromogenic substrates. S1 and S2 provide selective agents and additional growth factors (5).

According to the manufacturer, *S. pyogenes* colonies appear orange to red for ATCC 19615 strains and dark purple for another strain named “AR6426.” The AR6426 strain represents an uncommon phenotypic variant of *S. pyogenes,* with this designation being specific to CHROMagar (5).

*S. dysgalactiae* (Groups C and G) present as pink to purple colonies. Other streptococci may appear metallic blue or remain colorless. The medium is intended to inhibit Gram-negative organisms, most Gram-positive species, and yeasts (6).

To evaluate the diagnostic performance of CHROMagar StrepA, a three-phase experimental protocol was designed and implemented.

Previous studies have evaluated the performance of CHROMagar StrepA using both automated image analysis and manual interpretation approaches (7). Although automated interpretation systems based on artificial intelligence are available for chromogenic media, such technology was not utilized in the present study (7). All readings were performed visually by the same experienced technologist.

Given the descriptive nature of this diagnostic performance study, no inferential statistical tests were performed. Sensitivity, specificity, positive predictive value (PPV), and negative predictive value (NPV) were calculated using standard definitions.

### Phase 1: preliminary assessment using pure bacterial strains

The first phase focused on the preliminary evaluation of the medium using pure bacterial isolates. Bacterial strains, previously preserved in Schaedler semi-solid medium, were subcultured onto COL-S agar half-plates (Columbia agar supplemented with sheep blood). Following incubation at 37°C in a CO_₂_-enriched atmosphere for 24 h, colonies were subjected to identification by matrix-assisted laser desorption/ionization time-of-flight mass spectrometry (MALDI-TOF MS) to confirm strain purity. Once confirmed, the colonies were suspended in sterile physiological saline to achieve a turbidity equivalent to 0.5 McFarland. These suspensions were then inoculated onto both COL-S and CHROMagar StrepA media for comparative analysis, with COL-S serving as the reference control. Plates were incubated under the same conditions and examined at both 24 and 48 h.

**Fig 1:**
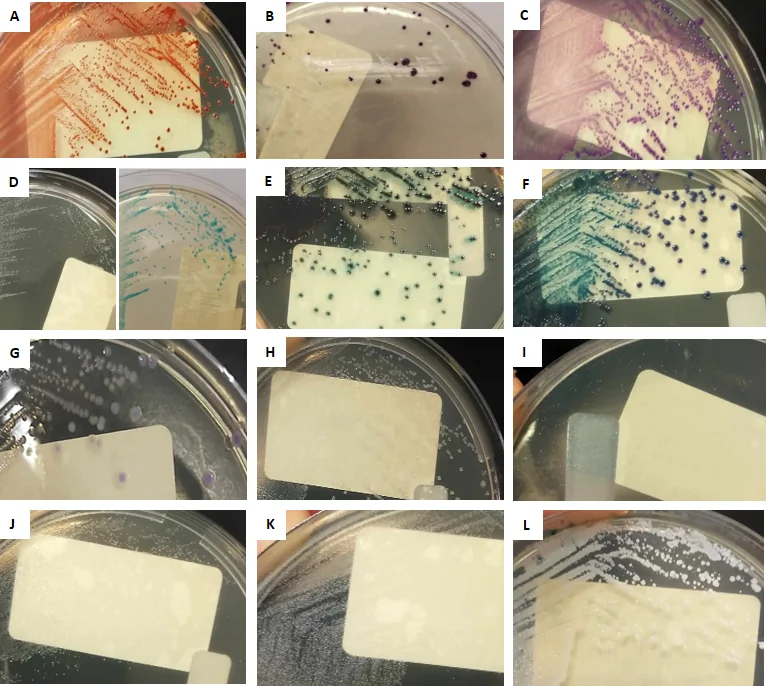
Appearance of different colonies on the CHROMagar StrepA medium (phase 1). (**A**) *Streptococcus pyogenes* ATCC 19615. (**B**) *Streptococcus pyogenes* AR6426. (**C**) *Streptococcus dysgalactiae*. (**D**) *Streptococcus intermedius* after 24 h left, and 48 h right. (**E**) *Enterococcus faecalis*. (**F**) Enterococcus faecium. (**G**) *Streptococcus agalactiae*. (**H**) *Streptococcus vestibularis*/*salivarius*. (**I**) *Streptococcus pneumoniae*. (**J**) *Streptococcus anginosus*. (**K**) *Streptococcus constellatus*. (**L**) *Staphylococcus epidermidis*.

This phase primarily included streptococcal strains commonly encountered in clinical practice. Additionally, a variety of Gram-positive cocci, Gram-negative cocci, and enterobacterial species were inoculated to assess the medium’s selectivity, particularly for beta-hemolytic streptococci. Each inoculated plate was systematically photographed to document and analyze colony morphology and coloration (Fig. 1).

### Phase 2: retrospective comparison with standard culture

The second phase involved a retrospective study utilizing throat swab samples previously confirmed as positive for beta-hemolytic streptococci. The positive specimens, collected using nylon flocked swabs (BD ESwab, Copan Italia S.p.A., Brescia, Italy) and preserved in liquid eSwab medium, were vortexed to ensure homogenization. A 50-µL aliquot of each sample was plated onto both COL-S and CHROMagar StrepA media. Following inoculation, the plates were incubated at 37°C in a CO_₂_-rich environment. Colony morphology and color were assessed at 24 and 48 h, and all colonies were identified using MALDI-TOF MS.

To ensure the integrity of the experimental process, a daily negative control was included by inoculating CHROMagar StrepA plates with sterile physiological saline. This step verified the absence of contamination during laboratory handling.

### Phase 3: routine diagnostic implementation

The third phase aimed to assess the performance of CHROMagar StrepA under routine diagnostic laboratory conditions. All throat swab samples received by the laboratory during the study period were analyzed in parallel using three methods: conventional culture on COL-S medium, CHROMagar StrepA, and a rapid antigen detection test (OSOM Strep A Test). After incubation under standard conditions (37°C, 5% CO_₂_), plates were examined at 24 and 48 h. All colonies obtained were subjected to MALDI-TOF MS identification to ensure accurate organism classification.

## RESULTS

### Phase 1: evaluation of the chromogenic medium using pure strains

Following the subculture of pure strains, colony color and morphology were assessed and compared with the manufacturer’s specifications.

*S. pyogenes* colonies were medium-sized and predominantly orange-red, consistent with the manufacturer’s indication for ATCC 19615 strains. A single isolate showed a dark purple to brown coloration, aligning with the profile described for AR6426 strains. Among 39 *S. pyogenes* isolates grown on CHROMagar StrepA, 38 showed typical orange-red colonies, and one showed the darker variant (Fig. 1A and B). The AR6426 strain is unlikely to significantly impact routine diagnostic performance due to its rarity. Although this pigmentation may appear atypical, it did not prevent identification by MALDI-TOF MS and remained distinguishable from other organisms growing on the medium, including *S. dysgalactiae*, which exhibited a lighter mauve coloration.

*S. dysgalactiae* also grew well, producing medium-sized colonies with pink to mauve hues (Fig. 1C).

Enterococci grew well with distinct colony colors. *E. faecalis* formed medium-sized, black colonies with a dark green halo, whereas *E. faecium* produced dark blue colonies (Fig. 1E and F).

*Streptococcus parasanguinis* formed small, dark turquoise-blue colonies after 24 h, and with no further change observed at 48 h.

*Streptococcus intermedius* formed small, initially transparent colonies at 24 h, which developed a turquoise-blue coloration after 48 h of incubation (Fig. 1D).

*S. constellatus* and *S. anginosus* exhibited transparent to gray colonies at both 24 and 48 h (Fig. 1J and K), indicating that under these conditions, CHROMagar StrepA does not allow reliable differentiation between these species.

*S. agalactiae* colonies appeared transparent with a light mauve center after 24 h of incubation (Fig. 1G), whereas *S. vestibularis* and *S. salivarius* showed small, whitish, transparent colonies across both incubation times (Fig. 1H).

*S. pneumoniae* showed minimal growth, forming very small, transparent colonies, likely due to its requirement for blood-derived growth factors (Fig. 1I).

Unexpectedly, growth was observed for two species not expected to grow on this medium. *Granulicatella adiacens* formed small, dry, strongly adherent colonies with coloration ranging from white to yellow, while *Staphylococcus epidermidis* produced small white colonies at 24 h, which increased to medium-sized colonies after 48 h (Fig. 1L). Although CHROMagar StrepA is designed to inhibit non-streptococcal Gram-positive bacteria, growth of these species was observed. In contrast, Gram-negative bacteria and yeasts were effectively inhibited, as expected.

To investigate *S. epidermidis* growth, antibiotic susceptibility testing was conducted for *S. epidermidis*, *S. aureus*, and MRSA. Results did not clarify the mechanism underlying this selective growth anomaly. A growth factor inherent to the medium may be responsible.

### Phase 2: retrospective comparison with COL-S medium

Twenty-two archived throat swab samples previously confirmed as positive for beta-hemolytic streptococci (18 GAS and 4 Groups C and G) were cultured on both COL-S and CHROMagar StrepA using a 50-µL inoculum from eSwab medium. All isolates were confirmed by MALDI-TOF.

During this phase, analysis of the 22 throat swabs showed systematic positivity for group A, C, and G streptococci on both culture media. Quantitative comparison revealed no major differences in bacterial load between media, though minor variations were noted at lower bacterial concentrations (1+). Both media supported comparable growth (Table 1).

**TABLE 1:** Semi-quantitative comparison of colony growth on COL-S and CHROMagar StrepA media using four *S*. *dysgalactiae* and eighteen *S. pyogenes* at identical concentrations onto both media and incubated for 24 h (phase 2)^*a*^

N°	Pathogen	COL-S	CHROMagar StrepA
1	*S. dysgalactiae*	1+	Rare
2	*S. dysgalactiae*	3+	3+
3	*S. dysgalactiae*	4+	4+
4	*S. dysgalactiae*	2+	2+
5	*S. pyogenes*	1+	Rare
6	*S. pyogenes*	1+	1+
7	*S. pyogenes*	4+	4+
8	*S. pyogenes*	1+	1+
9	*S. pyogenes*	2+	2+
10	*S. pyogenes*	3+	3+
11	*S. pyogenes*	3+	3+
12	*S. pyogenes*	3+	3+
13	*S. pyogenes*	3+	3+
14	*S. pyogenes*	3+	3+
15	*S. pyogenes*	2+	2+
16	*S. pyogenes*	1+	1+
17	*S. pyogenes*	2+	2+
18	*S. pyogenes*	3+	3+
19	*S. pyogenes*	4+	4+
20	*S. pyogenes*	3+	3+
21	*S. pyogenes*	2+	2+
22	*S. pyogenes*	3+	3+

^*a*^ Semi-quantitative colony growth was graded as 1+ (scant growth), 2+ (moderate growth), 3+ (heavy growth), and 4+ (very heavy growth).

**Fig 2:**
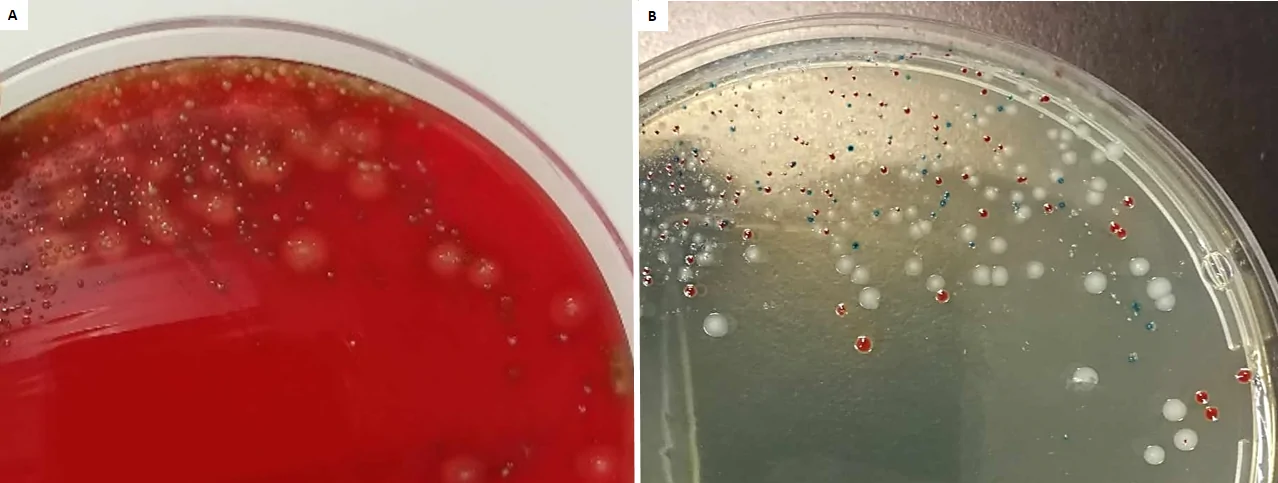
Colony appearance after 24 h for the same sample inoculated with 50 µL of a known positive streptococcal strain (phase 2). (**A**) Sample inoculated on COL-S medium. (**B**) Sample inoculated on CHROMagar StrepA medium, where *Streptococcus pyogenes* appears orange-red, *Streptococcus parasanguinis* appears blue, *Streptococcus salivarius* appears as large white colonies, and *Granulicatella adiacens* appears as fine, white-translucent colonies.

Visually, colony differentiation was markedly easier on CHROMagar StrepA, especially when colony counts were low. On COL-S, colonies from Groups A, C, and G appeared similarly, transparent and beta-hemolytic, while the chromogenic medium provided distinct visual cues, expediting detection (Fig. 2).

A negative control (sterile physiological saline) was inoculated daily onto the chromogenic medium to monitor the contamination. All controls remained sterile after 24 and 48 h.

### Phases 3: comparative performance in routine diagnostics

A total of 78 throat swab specimens were analyzed between 11 March and 10 April 2024. Patient ages ranged from 1 to 79 years, with the majority of samples obtained from individuals aged 15 to 64 years. Female patients accounted for 56% of the study population. Overall, 97% of the specimens were collected from outpatients, while 3% originated from the hospital emergency department. This distribution is consistent with expected clinical practice, as hospitalized patients are generally subject to strict infection prevention and control measures that limit the indication for routine pharyngeal sampling. In contrast, outpatients more frequently present with acute upper respiratory symptoms, resulting in higher testing rates in this population.

Among these 78 collected samples, 21 were positive for GAS on both COL-S and CHROMagar StrepA media, demonstrating perfect concordance between these two culture media for GAS detection. Six samples were positive for *S. dysgalactiae* on COL-S, whereas only four were detected on CHROMagar StrepA. The two samples not detected on CHROMagar StrepA corresponded to specimens with low bacterial loads. Finally, 51 samples were negative for both pathogens on both media.

In contrast, the rapid diagnostic test specific for GAS identified only 17 of the 21 culture-positive samples on COL-S. Moreover, one positive result was observed in a sample that was negative by culture, corresponding to one false positive and four false negatives. These false-positive results may be explained by the persistence of streptococcal antigens after bacterial death, allowing detection by the rapid test while preventing bacterial growth in culture. Further studies are required to confirm this hypothesis.

As shown in Table 2, the chromogenic medium demonstrated 100% sensitivity, specificity, PPV, and NPV of 100% for GAS detection compared with the reference medium COL-S. In contrast, the rapid diagnostic test exhibited a markedly lower sensitivity of 81%.

**TABLE 2:** Performance of CHROMagar StrepA and a rapid antigen detection test compared with COL-S culture for the detection of *S. pyogenes* from 78 consecutive clinical throat swabs collected prospectively (phase 3)^*a*^

Method	Phase	Total samples	TP	FP	TN	FN	Sensitivity (%)	Specificity (%)	PPV (%)	NPV (%)
CHROMagar StrepA	Phase 3	78	21	0	51	0	100	100	100	100
Rapid Test StrepA	Phase 3	78	17	1	56	4	81	98	94	93

^*a*^ True positives (TPs) represent samples correctly identified as positive, false positives (FPs) represent samples incorrectly identified as positive, true negatives (TNs) represent samples correctly identified as negative, and false negatives (FNs) represent samples incorrectly identified as negative. These values are presented alongside sensitivity, specificity, positive predictive value (PPV), and negative predictive value (NPV).

Regarding *S. dysgalactiae*, the limited number of positive samples reduces the robustness of the statistical analysis. The diagnostic performance, reported in Table 3, showed a sensitivity of 97%, specificity of 100%, PPV of 100%, and an NPV of 96%. However, these results should be interpreted with caution and require confirmation in larger-scale studies to validate their reliability.

**TABLE 3:** Performance of CHROMagar StrepA compared with COL-S culture for the detection of *S. dysgalactiae* from 78 consecutive clinical throat swabs collected prospectively (phase 3)^*a*^

Method	Phase	Total samples	TP	FP	TN	FN	Sensitivity (%)	Specificity (%)	PPV (%)	NPV (%)
CHROMagar StrepA	Phase 3	78	4	0	51	2	67	100	100	96

^*a*^ True positives (TPs), false positives (FPs), true negatives (TNs), and false negatives (FNs) are shown alongside sensitivity, specificity, positive predictive value (PPV), and negative predictive value (NPV). This table should be interpreted with caution due to the low number of true positive samples.

It should be noted that phase 2 data were not included in the performance calculations. Indeed, the final diagnostic performance measures were derived exclusively from phase three clinical samples to avoid selection bias introduced by the inclusion of pre-characterized positive isolates. Phase 2 samples were used solely for semi-quantitative interpretation purposes.

## DISCUSSION

The findings of this study confirm the diagnostic value of CHROMagar StrepA as a robust chromogenic medium for the detection of *S. pyogenes*. Compared with the conventional COL-S culture medium and the rapid antigen detection test, CHROMagar StrepA demonstrated excellent specificity (100%) and sensitivity (100%), making it a reliable tool for clinical microbiology laboratories to detect GAS. The perfect sensitivity and specificity observed during phase 3 should be interpreted with caution due to the limited sample size. Larger-scale prospective studies are required to confirm these findings in routine clinical practice. Such studies would also allow a more accurate evaluation of *S. dysgalactiae* performance, given the low number of positive samples for this pathogen in the present study. Effectively, Group C and Group G streptococci were identified among the isolates, but their numbers were insufficient to allow meaningful statistical analysis. These data are therefore presented descriptively only, as the primary objective of the study was to evaluate the performance of CHROMagar StrepA for the detection of *S. pyogenes*.

GAS isolates produced a characteristic chromogenic reaction within 24 h of incubation. However, some non-GAS strains required prolonged incubation up to 48 h to exhibit definitive coloration. This suggests that although a 24-h incubation is sufficient for most isolates, delayed chromogenic expression may occur in rare cases, potentially related to strain-specific metabolic variability. Importantly, extending incubation to 48 h did not yield additional false-positive or false-negative results in this study, indicating that neither specificity nor sensitivity was affected. Based on these observations, a 24-h incubation appears adequate for routine use.

Although the incubation time was identical for CHROMagar StrepA and COL-S (24 h), the chromogenic medium enables faster presumptive interpretation by allowing direct visual discrimination of colonies, as shown in Fig. 2. This feature was particularly advantageous in specimens with low bacterial loads, where beta-hemolysis on conventional media can be difficult to detect. In contrast, COL-S plates often required closer examination to distinguish *S. pyogenes* from other beta-hemolytic streptococci, resulting in longer hands-on reading times. This reduced hands-on interpretation time and improved screening efficiency during routine laboratory workflow. It should also be noted that CHROMagar StrepA is more expensive than standard culture media, such as COL-S. Its higher cost could potentially be justified by its operational advantages: easier detection, reduced need for extensive downstream testing, and better colony differentiation. In laboratories with limited staff resources or where rapid interpretation is required, this medium can effectively improve workflow efficiency and detection rates, thereby partially offsetting its initial cost.

When compared with the rapid antigen detection test, CHROMagar StrepA clearly outperforms in terms of sensitivity (100% vs 81%) and negative predictive value (100% vs 93%). However, this improvement in diagnostic accuracy comes at the cost of longer turnaround time. While the rapid test delivers results within minutes, CHROMagar StrepA requires 24 h of incubation. Therefore, although the chromogenic medium is more sensitive, it is not suited for immediate decision-making in urgent clinical settings. Its application is more appropriate in routine diagnostics, particularly where confirmatory culture remains essential.

For *S. dysgalactiae,* two isolates were negative on CHROMagar StrepA despite being positive on the reference culture, suggesting reduced sensitivity of CHROMagar StrepA for non-GAS beta-hemolytic streptococci. It should be noted that CHROMagar StrepA is specifically designed for the detection of *S. pyogenes* and is not intended for the recovery or identification of *S. dysgalactiae*. Laboratories interested in isolating both *S. pyogenes* and *S. dysgalactiae* may consider alternative chromogenic media, such as CHROMagar ACG.

Despite its advantages, certain limitations must be considered, such as the unexpected growth of *Staphylococcus epidermidis*, a coagulase-negative *Staphylococcus* theoretically excluded by the medium’s selective formulation, suggesting a potential selectivity issue warranting further investigation.

A limitation of this study is that all culture plates were read by a single technologist, and interpretations were not performed in a blinded manner. While this approach ensured consistency in reading criteria, it did not allow assessment of inter-operator variability and may have introduced interpretive bias. Future evaluations should incorporate blinded readings by multiple technologists to better assess reproducibility under routine laboratory conditions.

In conclusion, CHROMagar StrepA demonstrated high diagnostic performance for the detection of *Streptococcus pyogenes* in throat cultures, with 100% sensitivity and specificity and clear visual differentiation of colonies. Although it does not replace rapid antigen tests in time-critical situations, it is a reliable culture-based approach for confirming streptococcal infections, particularly in specimens with low bacterial load. Its ease of interpretation may facilitate routine laboratory workflow, despite a higher cost compared with conventional COL-S medium. Limitations include reduced selectivity for certain non-GAS streptococci and occasional growth of non-target organisms. Further large-scale studies in diverse clinical settings are required to confirm these findings and to refine the role of CHROMagar StrepA in diagnostic strategies.
